# Survival after thermal ablation versus wedge resection for stage I non-small cell lung cancer < 1 cm and 1 to 2 cm: evidence from the US SEER database

**DOI:** 10.1186/s40644-024-00733-4

**Published:** 2024-07-11

**Authors:** Shelly Yim, Wei Chan Lin, Jung Sen Liu, Ming Hong Yen

**Affiliations:** 1 Division of Chest Surgery, Department of Surgery, Cathay General Hospital, No. 280, Sec. 4, Renai Road, Daan District, Taipei, 106 Taiwan; 2 Department of Radiology, Cathay General Hospital, No. 280, Sec. 4, Renai Road, Daan District, Taipei, 106 Taiwan; 3 School of Medicine, Fu-Jen Catholic University, No. 69, Guizi Road, Taishan District, New Taipei City, 22241 Taiwan

**Keywords:** Thermal ablation, Wedge resection, Non-small cell lung cancer (NSCLC), Early stage, Surveillance Epidemiology and End results (SEER)

## Abstract

**Background:**

This study compared the survival outcomes after thermal ablation versus wedge resection in patients with stage I non-small cell lung cancer (NSCLC) ≤ 2 cm.

**Methods:**

Data from the United States (US) National Cancer Institute Surveillance Epidemiology and End Results (SEER) database from 2004 to 2019 were retrospectively analyzed. Patients with stage I NSCLC and lesions ≤ 2 cm who received thermal ablation or wedge resection were included. Patients who received chemotherapy or radiotherapy were excluded. Propensity-score matching (PSM) was applied to balance the baseline characteristics between patients who underwent the two procedures.

**Results:**

Univariate and Cox regression analyses were performed to determine the associations between study variables, overall survival (OS), and cancer-specific survival (CSS). After PSM, 328 patients remained for analysis. Multivariable Cox regression analysis revealed, compared to wedge resection, thermal ablation was significantly associated with a greater risk of poor OS (adjusted HR [aHR]: 1.34, 95% CI: 1.09–1.63, *p* = 0.004) but not CSS (aHR: 1.28, 95% CI: 0.96–1.71, *p* = 0.094). In stratified analyses, no significant differences were observed with respect to OS and CSS between the two procedures regardless of histology and grade. In patients with tumor size 1 to 2 cm, compared to wedge resection, thermal ablation was significantly associated with a higher risk of poor OS (aHR: 1.35, 95% CI: 1.10–1.66, *p* = 0.004). In contrast, no significant difference was found on OS and CSS between thermal ablation and wedge resection among those with tumor size < 1 cm.

**Conclusions:**

In patients with stage I NSCLC and tumor size < 1 cm, thermal ablation has similar OS and CSS with wedge resection.

## Background

Lung cancer, causing an estimated 1.6 million deaths globally each year, is a leading cause of cancer mortality worldwide [1]. Approximately 85% of lung cancers are non-small cell lung cancer (NSCLC), primarily adenocarcinoma and squamous cell carcinoma [2]. Lung cancer incidence increases with age, peaking between 65 and 84 years old [3]. The 5-year survival rate for NSCLC varies greatly by stage, ranging from 68% in stage IB to only 0-10% in stages IVa-IVb [4]. NSCLC often remains undiagnosed until it reaches an advanced stage [5]. Recent advancements in thin-section and low-dose computed tomography (CT) screening have made early-stage lung cancer detection more common, allowing precise assessment of lesion size and locations [6, 7].

Unnecessary removal of healthy lung tissue can markedly affect quality of life [8]. Older patients often benefit from less invasive surgeries that conserve healthy lung tissue. For certain patients, wedge resection, as compared to standard lobectomy, might preserve more lung parenchyma and thus better maintain lung function [9–12]. However, there are concerns that sublobar resection, especially wedge resection, is associated with a higher recurrence rate than lobectomy [13].

Percutaneous thermal ablation is increasingly used in early-stage NSCLC [14]. This method, involving either radiofrequency ablation (RFA) or microwave ablation, heats pathologic lung tissue to lethal temperatures, destroying the tumor and adjacent potentially malignant tissue [15]. An important advantage of thermal ablation is that surrounding tissue incurs minimal damage. A study by Zeng et al. compared stage I NSCLC treatments, finding thermal ablation and wedge resection led to similar overall survival (OS) and cancer-specific survival in patients aged over 75 years [16]. However, the study did not compare the procedures based on tumor size, leaving their effectiveness in smaller tumors uncertain.

To fill this knowledge gap, the purpose of this study was to compare the outcomes of wedge resection and thermal ablation in persons with NSCLC tumor sizes < 2 cm using data from a US national database.

## Methods

### Study design and data source

Data from the US National Cancer Institute (NCI) Surveillance Epidemiology and End Results (SEER) database from 2004 to 2019 were retrospectively reviewed. The SEER project was begun in 1973 in the US as a population-based registry of cancer and involves about one-tenth of the US population. The patient sample of this study was selected from de-identified patients in 17 SEER registries (SEER*Stat Database: Incidence – SEER Research Plus Data, 17 Registries, Nov 2021 Sub (2000–2019)). The data include no personal identifiers, and were submitted to the NCI electronically, and thus allowing the data to be used in relevant medical research [17, 18]. Because this study analyzed secondary data from the publicly accessible database, no patients were involved directly. The study was approved by the local Ethics Committee and Institutional Review Board to use de-identified data, including patient clinicopathological features, tumor histology, tumor size, timing, and type of first-course treatment, and outcomes.

### Patient selection criteria

The inclusion criteria were: (1) patients with NSCLC identified using tumor histology ICD-O-3 codes 8010, 8012, 8013, 8020, 8046, 8050–8052, 8070–8078, 8140, 8141, 8143, 8147, 8250–8255, 8260, 8310, 8430, 8480, 8481, 8490, 8560, and 8570–8575; (2) the first cancer of life; (3) tumor size ≤ 2 cm; and (4) underwent thermal ablation or wedge resection. In the SEER database, thermal ablation includes laser ablation, cautery, and fulguration. Exclusion criteria were: (1) missing OS or CCS information; (2) patients survived < 1 month after the procedure was performed; (3) unknown T, N, or M stage, or T0 and Tis stage; (4) with M1 stage disease; (5) lymph node-positive; and (6) patients received chemotherapy or radiotherapy. Eligible patients were divided into a thermal ablation group and a wedge resection group.

### Study variables and endpoints

The study endpoints were OS and CSS. Demographic data included age at diagnosis, sex, race/ethnicity (white, black, or other), year of diagnosis (2004–2009 and 2010–2019), and marital status. The tumor-related characteristics included tumor side (left or right), site (upper lobe, middle lobe, lower lobe, unspecified), tumor size (< 1 cm or 1 to 2 cm), tumor histology (squamous cell carcinoma, adenocarcinoma, or other), and tumor grade (I/II, III/IV, or unknown).

### Statistical analysis

Categorical data were presented as number (n) and percentage (%), and compared with the chi-squared test or Fisher’s exact test, as appropriate. Continuous data without a normal distribution were presented as the median and interquartile range (IQR), and compared with the Wilcoxon rank sum test. Propensity score matching (PSM) was performed using 1:1 nearest neighbor matching to obtain matched pairs between the thermal ablation and wedge resection groups. Variables having a value of *p* < 0.001 between the comparison groups were used in PSM. Cox proportional hazard regression analyses were conducted to estimate the hazard ratio (HR) and 95% confidence interval (CI) of the associations between study variables, OS, and CSS. Variables with a value of *p* < 0.05 in the univariate analysis were entered into the multivariate analyses. Kaplan–Meier survival curves were plotted according to the treatment modality. All analyses were 2-sided, and a value of *p* < 0.05 was regarded as statistically significant. Data management and statistical analyses were performed using SAS version 9.4 software (SAS Institute, Inc.).

## Results

**Fig. 1:**
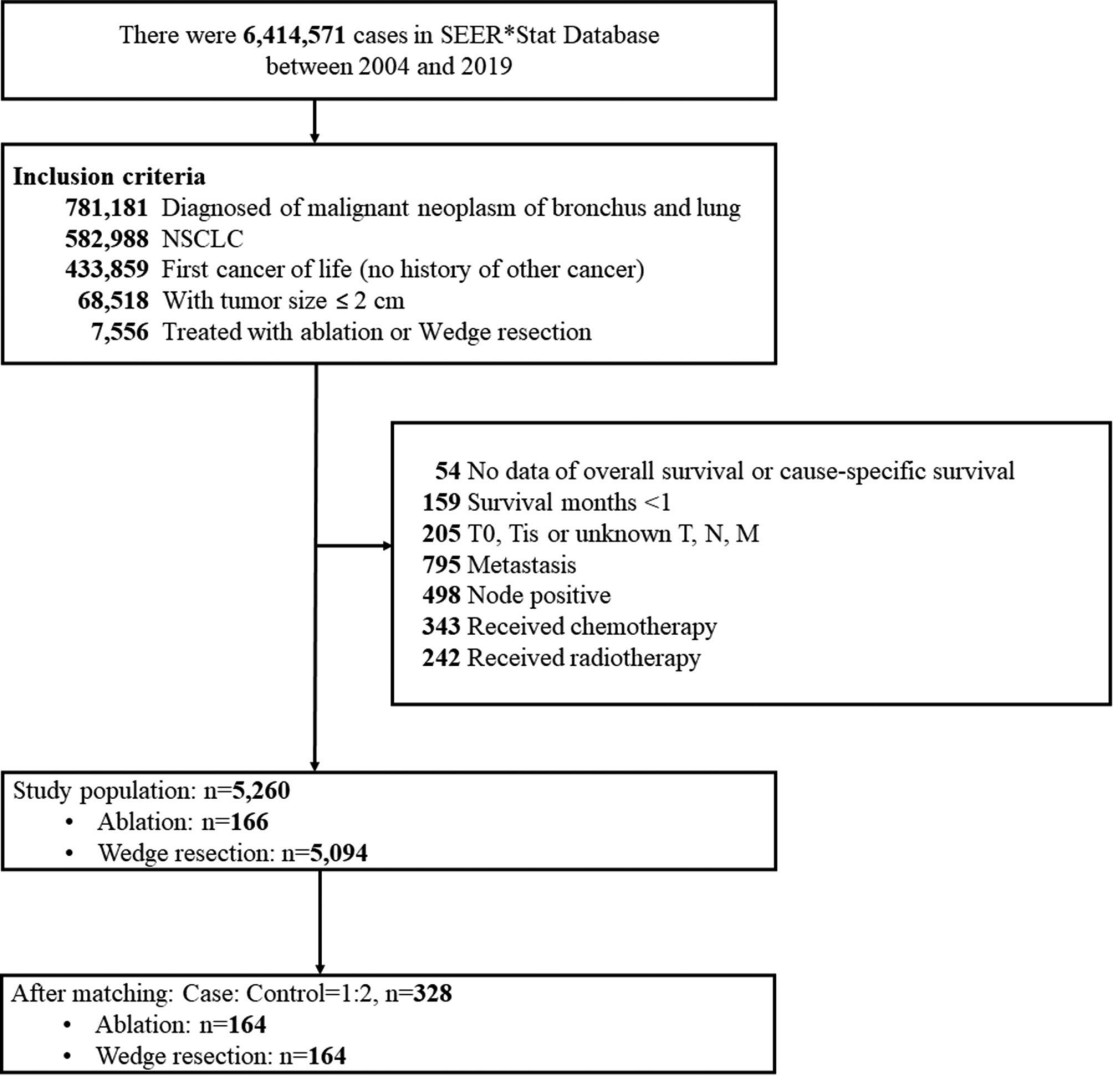
Flow Diagram of study selection process

The flow diagram of the patient selection process is depicted in Fig. 1. Overall, there were 6,414,571 patients in the SEER database from 2004 to 2019. Of this population, 781,181 were diagnosed with a malignant lesion of the bronchus and lung. Further examination of the data retrieved 7,556 patients with NSCLC and a tumor size ≤ 2 cm, received thermal ablation or wedge resection as the primary treatment. After excluding patients with missing survival data, survival < 1 month, T0/Tis or unknown T, N, or M stage, metastatic disease, lymph node-positive disease, or had received chemotherapy or radiotherapy, 5,260 patients were identified and included in the analysis. Of the patients, 3.2% received thermal ablation. After 1:1 PSM, there remained 328 patients in the analytic sample (Fig. 1).

### Characteristics of patients who received thermal ablation and wedge resection

Patient baseline characteristics are summarized in Table 1. The median age of all patients was 70 years, and more than half of the patients were females (58.6%). The majority of patients were White (86.9%), diagnosed during the period from 2010 to 2019 (65.7%), and were married (55.8%). More than half of the patients had an adenocarcinoma (70.2%), a grade I/II (63.6%) tumor, a tumor size of 1 to 2 cm (85.2%), and the tumor located in an upper lobe (64.8%).

**Table 1:** Baseline characteristics of patients with stage I NSCLC ≤ 2 cm

Characteristics	Before PSM				After 1:1 PSM			
	Total (*N* = 5,260)	Ablation (*n* = 166)	Wedge resection (*n* = 5,094)	*p*	Total (*n* = 328)	Ablation (*n* = 164)	Wedge resection (*n* = 164)	*p*
**Demography**								
**Age, years**	70 (63–76)	73 (67–81)	70 (63–76)	**< 0.001**				1.000
< 60	810 (15.4)	15 (9.0)	795 (15.6)	**< 0.001**	30 (9.1)	15 (9.1)	15 (9.1)	
60–64	686 (13.0)	16 (9.6)	670 (13.2)		33 (10.1)	16 (9.8)	17 (10.4)	
65–69	1035 (19.7)	24 (14.5)	1011 (19.8)		47 (14.3)	24 (14.6)	23 (14.0)	
70–74	1101 (20.9)	36 (21.7)	1065 (20.9)		72 (22.0)	36 (22.0)	36 (22.0)	
75–79	906 (17.2)	26 (15.7)	880 (17.3)		52 (15.9)	26 (15.9)	26 (15.9)	
≥ 80	722 (13.7)	49 (29.5)	673 (13.2)		94 (28.7)	47 (28.7)	47 (28.7)	
**Sex**				0.713				0.654
Male	2177 (41.4)	71 (42.8)	2106 (41.3)		136 (41.5)	70 (42.7)	66 (40.2)	
Female	3083 (58.6)	95 (57.2)	2988 (58.7)		192 (58.5)	94 (57.3)	98 (59.8)	
**Race**				0.719				0.824
White	4555 (86.9)	142 (86.1)	4413 (87.0)		284 (86.9)	140 (85.9)	144 (87.8)	
Black	397 (7.6)	15 (9.1)	382 (7.5)		27 (8.3)	15 (9.2)	12 (7.3)	
Other	288 (5.5)	8 (4.8)	280 (5.5)		16 (4.9)	8 (4.9)	8 (4.9)	
Unknown	20	1	19		1	1	0	
**Year of diagnosis**				**< 0.001**				0.912
2004 to 2009	1804 (34.3)	87 (52.4)	1717 (33.7)		169 (51.5)	85 (51.8)	84 (51.2)	
2010 to 2019	3456 (65.7)	79 (47.6)	3377 (66.3)		159 (48.5)	79 (48.2)	80 (48.8)	
**Marital status**				**0.020**				0.273
Single	593 (11.8)	21 (13.0)	572 (11.7)		34 (10.7)	21 (13.2)	13 (8.2)	
Married	2814 (55.8)	73 (45.3)	2741 (56.1)		154 (48.6)	72 (45.3)	82 (51.9)	
Separated/divorced/widowed	1637 (32.5)	67 (41.6)	1570 (32.2)		129 (40.7)	66 (41.5)	63 (39.9)	
Unknown	216	5	211		11	5	6	
**Tumor-related**								
**Histology**				**< 0.001**				1.000
Squamous cell carcinoma	1284 (24.4)	44 (26.5)	1240 (24.3)		88 (26.8)	44 (26.8)	44 (26.8)	
Adenocarcinoma	3692 (70.2)	89 (53.6)	3603 (70.7)		178 (54.3)	89 (54.3)	89 (54.3)	
Other	284 (5.4)	33 (19.9)	251 (4.9)		62 (18.9)	31 (18.9)	31 (18.9)	
**Grade**				**< 0.001**				0.989
I / II	3345 (63.6)	43 (25.9)	3302 (64.8)		86 (26.2)	43 (26.2)	43 (26.2)	
III / IV	1352 (25.7)	29 (17.5)	1323 (26.0)		59 (18.0)	29 (17.7)	30 (18.3)	
Unspecified	563 (10.7)	94 (56.6)	469 (9.2)		183 (55.8)	92 (56.1)	91 (55.5)	
**Tumor size, cm**				**0.005**				0.659
< 1	776 (14.8)	12 (7.2)	764 (15.0)		22 (6.7)	12 (7.3)	10 (6.1)	
1 to 2	4484 (85.2)	154 (92.8)	4330 (85.0)		306 (93.3)	152 (92.7)	154 (93.9)	
**Tumor side**				0.143				0.058
Right	2972 (56.5)	103 (62.0)	2869 (56.3)		185 (56.4)	101 (61.6)	84 (51.2)	
Left	2288 (43.5)	63 (38.0)	2225 (43.7)		143 (43.6)	63 (38.4)	80 (48.8)	
**Tumor Site**				0.341				0.511
Upper lobe	3363 (64.8)	97 (59.9)	3266 (64.9)		202 (62.9)	96 (60.0)	106 (65.8)	
Lower lobe	1618 (31.2)	59 (36.4)	1559 (31.0)		109 (34.0)	58 (36.3)	51 (31.7)	
Middle lobe	210 (4.0)	6 (3.7)	204 (4.1)		10 (3.1)	6 (3.8)	4 (2.5)	
Unspecified	69	4	65		7	4	3	

Categorical data are presented as number (%), and continuous data are presented as median and interquartile rage (IQR). Significant results are shown in bold

PSM, propensity-score matching; NSCLC, non-small cell lung cancer

As compared to patients who received wedge resection, those who received thermal ablation were older (median 73 vs. 70 years), had lower proportion who were diagnosed during the period from 2010 to 2019 (47.6% vs. 66.3%, *p* < 0.001), had a lower proportion who were married (45.3% vs. 56.1%, *p* = 0.020), a lower percentage of adenocarcinoma (53.6% vs. 70.7%, *p* < 0.001), and a lower percentage of tumor size < 1 cm (7.2% vs. 15.0%, *p* = 0.005). However, patients who received thermal ablation had a higher proportion of tumors with an unspecified grade (56.6% vs. 9.2%, *p* < 0.001). After PSM, differences in baseline characteristics between the two groups were gone (Table 1).

### Association between thermal ablation vs. wedge resection, OS, and CSS after PSM

**Fig. 2:**
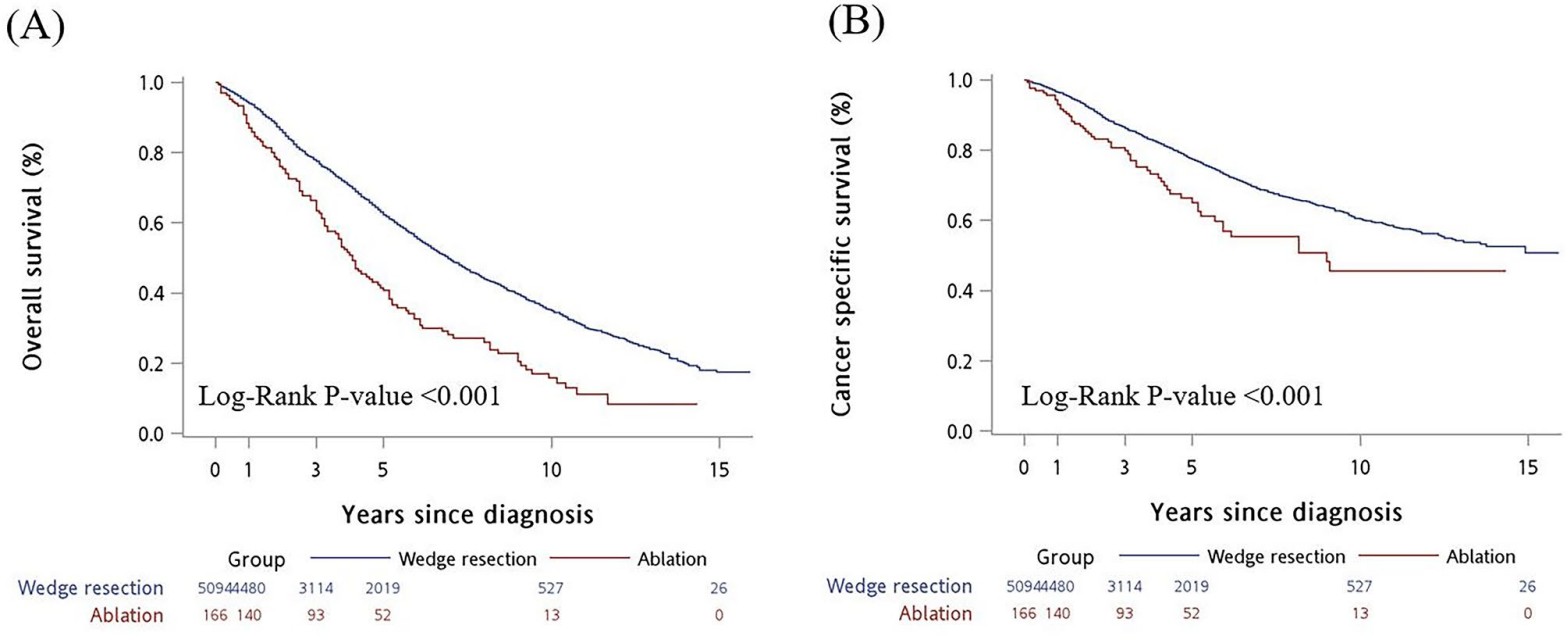
Kaplan–Meier survival curves were plotted according to treatment modality. (**A**) Overall survival. (**B**) Cancer specific survival

As shown in Fig. 2, patients who received thermal ablation had a poorer OS and CSS than those who received wedge resection (both log-rank *p* < 0.001).

Results of the univariate and multivariable Cox regression analysis of OS and CSS after PSM are shown in Table 2. After adjusting for relevant confounders, compared to wedge resection, thermal ablation was significantly associated with a greater risk of poor OS (adjusted HR [aHR]: 1.34, 95% CI: 1.09–1.63, *p* = 0.004) but not CSS (aHR: 1.28, 95% CI: 0.96–1.71, *p* = 0.094) (Table 2).

**Table 2:** Cox regression analysis of OS and CSS of patients with stage I NSCLC ≤ 2 cm, after PSM

Study variables	OS				CSS			
	HR (95% CI)	*p*	aHR (95% CI)	*p*	HR (95% CI)	*p*	aHR (95% CI)	*p*
Ablation vs. wedge resection	1.82 (1.51–2.20)	**< 0.001**	1.34 (1.09–1.63)	**0.004**	1.63 (1.24–2.15)	**< 0.001**	1.28 (0.96–1.71)	0.094
Age, years (vs. < 60)								
60–64	1.52 (1.28–1.80)	**< 0.001**	1.44 (1.21–1.72)	**< 0.001**	1.51 (1.20–1.90)	**< 0.001**	1.49 (1.18–1.88)	**0.001**
65–69	1.51 (1.29–1.77)	**< 0.001**	1.46 (1.24–1.72)	**< 0.001**	1.46 (1.18–1.80)	**< 0.001**	1.47 (1.18–1.82)	**0.001**
70–74	1.98 (1.70–2.30)	**< 0.001**	1.85 (1.58–2.17)	**< 0.001**	1.63 (1.33–2.01)	**< 0.001**	1.64 (1.32–2.03)	**< 0.001**
75–79	2.59 (2.23–3.02)	**< 0.001**	2.34 (1.99–2.74)	**< 0.001**	2.09 (1.69–2.58)	**< 0.001**	1.99 (1.60–2.47)	**< 0.001**
≥ 80	3.23 (2.77–3.76)	**< 0.001**	2.89 (2.46–3.40)	**< 0.001**	2.44 (1.97–3.02)	**< 0.001**	2.27 (1.81–2.84)	**< 0.001**
Sex (vs. female)								
Male	1.41 (1.30–1.52)	**< 0.001**	1.44 (1.32–1.57)	**< 0.001**	1.32 (1.18–1.47)	**< 0.001**	1.36 (1.20–1.53)	**< 0.001**
Race (vs. White)								
Black	0.89 (0.76–1.04)	0.130	0.94 (0.80–1.10)	0.429	1.13 (0.93–1.38)	0.221	1.19 (0.97–1.46)	0.103
Other	0.58 (0.46–0.71)	**< 0.001**	0.63 (0.51–0.79)	**< 0.001**	0.64 (0.47–0.86)	**0.003**	0.72 (0.53–0.97)	**0.033**
Year of diagnosis (vs. 2004 to 2009)								
2010 to 2019	0.76 (0.69–0.82)	**< 0.001**	0.83 (0.76–0.90)	**< 0.001**	0.69 (0.61–0.78)	**< 0.001**	0.76 (0.67–0.85)	**< 0.001**
Marital status (vs. single)								
Married	1.03 (0.90–1.18)	0.666	0.86 (0.75–0.99)	**0.034**	0.92 (0.76–1.10)	0.363	0.81 (0.67–0.98)	**0.033**
Separated/divorced/widowed	1.30 (1.13–1.50)	**< 0.001**	1.01 (0.87–1.17)	0.871	1.21 (1.00-1.47)	0.051	1.03 (0.84–1.25)	0.800
Histology (vs. squamous cell carcinoma)								
Adenocarcinoma	0.61 (0.56–0.67)	**< 0.001**	0.76 (0.69–0.83)	**< 0.001**	0.77 (0.67–0.88)	**< 0.001**	0.96 (0.83–1.10)	0.541
Others	1.13 (0.97–1.32)	0.112	1.06 (0.90–1.24)	0.478	1.58 (1.29–1.95)	**< 0.001**	1.42 (1.15–1.77)	**0.001**
Grade (vs. I / II)								
III / IV	1.50 (1.37–1.63)	**< 0.001**	1.28 (1.17–1.41)	**< 0.001**	1.59 (1.41–1.80)	**< 0.001**	1.37 (1.20–1.56)	**< 0.001**
Unspecified	1.18 (1.03–1.35)	**0.017**	1.13 (0.98–1.30)	0.099	1.17 (0.97–1.42)	0.109	1.04 (0.85–1.28)	0.719
Tumor size, cm (vs. < 1)								
1 to 2	1.44 (1.27–1.64)	**< 0.001**	1.21 (1.07–1.38)	**0.003**	1.48 (1.24–1.77)	**< 0.001**	1.23 (1.03–1.48)	**0.024**
Tumor side (vs. right)								
Left	1.06 (0.98–1.15)	0.153			1.03 (0.92–1.15)	0.633		
Tumor Site (vs. upper lobe)								
Lower lobe	0.98 (0.90–1.07)	0.719			1.07 (0.94–1.21)	0.299		
Middle lobe	0.98 (0.80–1.19)	0.817			1.18 (0.91–1.53)	0.207		

Significant results are shown in bold

NSCLC, non-small cell lung cancer; aHR, adjusted HR; CI, confidence interval; CSS, cancer-specific survival; HR, hazard ratio; OS, overall survival; PSM, propensity-score matching

### Stratified association between thermal ablation versus wedge resection, OS, and CSS

Results of multivariable Cox regression analyses of OS and CSS stratified by histology, tumor grade, and tumor size are summarized in Table 3. After adjustments, CSS was not significantly different between thermal ablation and wedge resection among all subgroups. In addition, OS was not significantly different between thermal ablation and wedge resection when stratified by histology or grade. Among patients with a tumor size < 1 cm, there were also no significant differences in OS between the two procedures; however, among patients with a tumor size 1 to 2 cm, thermal ablation was associated with a significantly higher risk of poor OS (aHR: 1.35, 95% CI: 1.10–1.66, *p* = 0.004) (Table 3).

**Table 3:** Stratified Cox regression analysis of OS and CSS in patients with stage I NSCLC ≤ 2 cm, after PSM

	Total	Thermal ablation vs. wedge resection					
		OS			CSS		
		Death	aHR (95% CI)	*p*	Event	aHR (95% CI)	*p*
**Histology** ^a^							
Adenocarcinoma	1236	696	1.34 (0.93–1.92)	0.117	302	1.47 (0.84–2.58)	0.176
Squamous cell carcinoma	3520	1470	1.25 (0.93–1.68)	0.134	782	0.89 (0.55–1.43)	0.623
**Grade** ^**b**^							
I / II	3181	1355	0.99 (0.66–1.49)	0.960	677	0.86 (0.46–1.61)	0.636
III / IV	1311	780	1.21 (0.80–1.83)	0.358	413	1.36 (0.79–2.33)	0.270
**Tumor size, cm** ^**c**^							
< 1	733	266	1.63 (0.64–4.17)	0.310	133	2.06 (0.59–7.12)	0.256
1 to 2	4298	2116	1.35 (1.10–1.66)	**0.004**	1076	1.29 (0.96–1.74)	0.090

NSCLC, non-small cell lung cancer; aHR, adjusted HR; CI, confidence interval; CSS, cancer-specific survival; HR, hazard ratio; OS, overall survival; PSM, propensity-score matching

^a^ Adjusted for age in category, sex, race, year, marital status, grade, and tumor size

^b^ Adjusted for age in category, sex, race, year, marital status, histology, and tumor size

^c^ Adjusted for age in category, sex, race, year, marital status, histology, and grade

## Discussion

This study investigated whether thermal ablation had an equivalent prognostic impact compared to wedge resection in patients with < 2 cm stage I NSCLC. The analyses revealed that patients who received thermal ablation had a higher risk of poor OS than wedge resection. However, according to the stratified analysis, OS and OSS between thermal ablation and wedge resection appear similar in any histology or grade. Finally, when stratified by tumor size, among patients with tumor size < 1 cm, no difference was found in OS and CSS between the two procedures. However, in patients with tumor size 1 to 2 cm, thermal ablation appears to have a higher risk of poor OS.

When NSCLC is diagnosed at an early stage during chest CT screening, sublobar resection is used as an alternative surgical strategy [19]. Segmentectomy shows better survival than wedge resection for tumors < 2 cm, with no difference for ≤ 1 cm tumors [20]. Sublobar resection matches lobectomy in survival and improves pulmonary function in peripheral stage IA NSCLC ≤ 2 cm [21]. Another study documented that for patients with NSCLC and peripheral tumors ≤ 2 cm, it is contentious whether segmentectomy or wedge resection is preferable [22]. Suzuki et al. find no significant difference in postoperative complications between segmentectomy and lobectomy, except for more air leakage in segmentectomy [23].

Although sublobar resections were developed to limit the functional impairments associated with lobectomy in early NSCLC, approximately 20% of patients are still ineligible for surgical procedures due to relatively severe comorbidities [24]. For instance, many individuals are ineligible for surgery because of advanced age or the presence of comorbidities such as pulmonary insufficiency or atherosclerosis [24]. An important advantage of thermal ablation over surgical resection is that it can destroy lung tumors by locally heating the lung parenchyma, while avoiding damage to surrounding normal lung tissue [25, 26]. Nevertheless, whether thermal ablation has equivalent long-term survival outcomes to wedge resection in early NSCLC is unclear and needs substantive evidence.

Using the SEER data from 2004 to 2019, our analytic results indicate that thermal ablation may be the best option for individuals with stage I NSCLC with a tumor size of 1 cm, since it is less invasive and led to no significantly different OS and CSS compared to wedge resection. As mentioned above, Zeng et al., querying the same database but with a shorter follow-up than our study, assessed the prognostic impact of thermal ablation versus wedge resection for stage I NSCLC with mixed tumor sizes [16]. It should be noted that no analysis was performed according to different tumor sizes in their report. Moreover, the result of their study was likely biased due to the lack of exclusion of chemotherapy and radiotherapy, thus limited interpretations.

The use of thermal ablation as an alternate therapy for patients who are not surgical candidates has increased because only one-third of stage I NSCLC patients are eligible for curative surgical resection [27]. It was previously believed that patients who received thermal ablation alone have a greater risk of local tumor recurrence because of insufficient ablation margins [28]. Inconsistent with our findings, a meta-analysis compared the relative safety and efficacy of thermal ablation and sublobar resection for treating stage I NSCLC and indicated that ablation was associated with shorter survival compared to surgery [29]. With regard to safety, it has been reported that thermal ablation for NSCLC, via percutaneous or bronchoscopic methods, can lead to complications such as pneumothorax, pleural effusion, pneumonia, and, rarely significant hemorrhage. However, bronchoscopic approaches generally show a safer profile with fewer adverse events [30, 31]. RFA shares similar complications, but these are typically temporary and manageable with appropriate treatments [32].

Each technology for thermal ablation has a benefit or detriment [33]. A previous single-center, retrospective study by Huang et al. evaluated 10-year OS, progression-free survival (PFS), and local control rates in patients with inoperable stage IA NSCLC who underwent CT-guided RFA [34]. The results showed that CT-guided RFA performed by a thoracic surgeon is a feasible, safe, and effective procedure for inoperable high-risk early-stage NSCLC, and should be considered as an alternative to sublobar resection. A recent study by Li et al. indicated that RFA, compared to no treatment, has better survival in patients with unresected stage IA NSCLC [35]. Another study by Streitparth et al. also highlighted the feasibility and safety of RFA in early, NSCLC [32].

### Strength and limitations

The study is inherently limited by its retrospective and observational nature. The SEER database lacks data on complications, cardiopulmonary function, performance status, and recurrence, precluding the incorporation of these into the analysis. In addition, SEER does not make it possible to know the ablation energy used, locations of tumors (central or peripheral), or patients’ baseline comorbidities. Furthermore, the notable low number of ablation cases across the entire population, as well as within specific subgroups analyzed, while potentially reflecting real-world scenarios, could pose a challenge to the reliability of the analytic results. Despite the abovementioned limitations, the present analyses utilized the latest data in a national cohort, and are likely to be robust under the implementation of PSM and multiple stratified analyses.

## Conclusions

For patients with stage I NSCLC and a tumor size of 1 to 2 cm, thermal ablation is associated with a higher risk of poor OS than wedge resection; however, in patients who had a tumor size < 1 cm, thermal ablation, and wedge resection show no difference on OS and CSS. Future prospective studies are still warranted to confirm the prognostic role of thermal ablation in this specific patient subgroup.

## Data Availability

The data generated in the present study may be requested from the corresponding author.

## Abbreviations

NSCLC: Non-small cell lung cancer
US: United States
SEER: Surveillance Epidemiology and End Results
OS: Overall survival
CSS: Cancer specific survival
CT: Computed tomography
HR: Hazard ratio

## References

[1] Thandra KC, Barsouk A, Saginala K, Aluru JS, Barsouk A (2021). Epidemiology of lung cancer. Contemp Oncol (Pozn).

[2] Herbst RS, Morgensztern D, Boshoff C (2018). The biology and management of non-small cell lung cancer. Nature.

[3] Shi J, Li D, Liang D, He Y (2021). Epidemiology and prognosis in young lung cancer patients aged under 45 years old in northern China. Sci Rep.

[4] Casal-Mouriño A, Ruano-Ravina A, Lorenzo-González M, Rodríguez-Martínez Á, Giraldo-Osorio A, Varela-Lema L (2021). Epidemiology of stage III lung cancer: frequency, diagnostic characteristics, and survival. Transl Lung Cancer Res.

[5] Saintigny P, Burger JA (2012). Recent advances in non-small cell lung cancer biology and clinical management. Discov Med.

[6] Gierada DS, Black WC, Chiles C, Pinsky PF, Yankelevitz DF (2020). Low-dose CT screening for Lung Cancer: evidence from 2 decades of study. Radiol Imaging Cancer.

[7] Postmus PE, Kerr KM, Oudkerk M, Senan S, Waller DA, Vansteenkiste J (2017). Early and locally advanced non-small-cell lung cancer (NSCLC): ESMO Clinical Practice guidelines for diagnosis, treatment and follow-up. Ann Oncol.

[8] Wang M, Herbst RS, Boshoff C (2021). Toward personalized treatment approaches for non-small-cell lung cancer. Nat Med.

[9] Alexander M, Lin E, Cheng H (2020). Leptomeningeal metastases in Non-small Cell Lung Cancer: optimal systemic management in NSCLC with and without driver mutations. Curr Treat Options Oncol.

[10] Shi Y, Wu S, Ma S, Lyu Y, Xu H, Deng L (2022). Comparison between Wedge Resection and Lobectomy/Segmentectomy for Early-Stage Non-small Cell Lung Cancer: a bayesian Meta-analysis and systematic review. Ann Surg Oncol.

[11] Song C, Lu Z, Li D, Pan S, Li N, Geng Q. Survival after wedge resection versus lobectomy for stage IA second primary NSCLC with previous lung cancer-directed surgery. Front Oncol. 2022;12(890033). 10.3389/fonc.2022.890033 10.3389/fonc.2022.890033 PMC9399676 36033457

[12] Wang P, Wang S, Liu Z, Sui X, Wang X, Li X, et al. Segmentectomy and Wedge Resection for Elderly patients with Stage I Non-small Cell Lung Cancer: a systematic review and Meta-analysis. J Clin Med. 2022;11(2). 10.3390/jcm11020294 10.3390/jcm11020294 PMC8782039 35053989

[13] Wu L, Zhao W, Chen T, Yang Y (2021). Surgical choice for patients with stage I non-small-cell lung cancer ≤ 2 cm: an analysis from surveillance, epidemiology, and end results database. J Cardiothorac Surg.

[14] Narsule CK, Sridhar P, Nair D, Gupta A, Oommen RG, Ebright MI (2017). Percutaneous thermal ablation for stage IA non-small cell lung cancer: long-term follow-up. J Thorac Dis.

[15] de Baere T, Tselikas L, Catena V, Buy X, Deschamps F, Palussière J (2016). Percutaneous thermal ablation of primary lung cancer. Diagn Interv Imaging.

[16] Zeng C, Lu J, Tian Y, Fu X. Thermal ablation Versus Wedge Resection for Stage I non-small cell Lung Cancer based on the Eighth Edition of the TNM classification: a Population Study of the US SEER database. Front Oncol. 2020;10(571684). 10.3389/fonc.2020.571684 10.3389/fonc.2020.571684 PMC7591765 33154946

[17] Goldwasser DL (2017). Estimation of the tumor size at cure threshold among aggressive non-small cell lung cancers (NSCLCs): evidence from the surveillance, epidemiology, and end results (SEER) program and the national lung screening trial (NLST). Int J Cancer.

[18] Polednak AP (2009). Lung cancer incidence trends by histologic type in areas of California vs. other areas in the Surveillance, Epidemiology and End results Program. Cancer Epidemiol.

[19] Sakurai H, Asamura H (2014). Sublobar resection for early-stage lung cancer. Transl Lung Cancer Res.

[20] Lei X, Zhou N, Zhang H, Li T, Ren F, Zhang B, et al. Lobe-Specific Analysis of Sublobar Lung Resection for NSCLC Patients with Tumors ≤ 2 cm. Cancers (Basel). 2022;14(13). 10.3390/cancers14133265 10.3390/cancers14133265 PMC9265391 35805037

[21] Altorki N, Wang X, Kozono D, Watt C, Landrenau R, Wigle D (2023). Lobar or Sublobar Resection for Peripheral Stage IA Non-small-cell Lung Cancer. N Engl J Med.

[22] Godfrey CM, Marmor HN, Lambright ES, Grogan EL (2022). Minimally invasive and Sublobar resections for Lung Cancer. Surg Clin North Am.

[23] Suzuki K, Saji H, Aokage K, Watanabe SI, Okada M, Mizusawa J (2019). Comparison of pulmonary segmentectomy and lobectomy: safety results of a randomized trial. J Thorac Cardiovasc Surg.

[24] Hamilton G, Rath B, Ulsperger E (2016). A review of the role of surgery for small cell lung cancer and the potential prognostic value of enumeration of circulating tumor cells. Eur J Surg Oncol.

[25] Benet J, Toffart AC, Brichon PY, Chollier T, Ruckly S, Villa J, et al. Survival of clinical stage III NSCLC according to therapeutic strategy: relevance of the tumor board decision in the era of immunotherapy. Cancer Treat Res Commun. 2022;30(100508). 10.1016/j.ctarc.2021.100508 10.1016/j.ctarc.2021.100508 35033834

[26] Kong F, Wang C, Li Y, Li X (2021). Advances in study of the sequence of lung tumor biopsy and thermal ablation. Thorac Cancer.

[27] Ridge CA, Solomon SB, Thornton RH (2014). Thermal ablation of stage I non-small cell lung carcinoma. Semin Intervent Radiol.

[28] Ghosn M, Solomon SB. Current management of Oligometastatic Lung Cancer and Future perspectives: results of thermal ablation as a local ablative therapy. Cancers (Basel). 2021;13(20). 10.3390/cancers13205202 10.3390/cancers13205202 PMC8534236 34680348

[29] Li Y, Yang F, Huang YY, Wang T (2022). Sublobar resection versus ablation for stage I non-small-cell lung cancer: a meta-analysis. J Cardiothorac Surg.

[30] Liu R, Shen Q, Lu H (2022). The efficacy and safety of thermal ablation for patients with lung malignancy: a meta-analysis of 12 studies in China. J Cardiothorac Surg.

[31] Rangamuwa K, Leong T, Weeden C, Asselin-Labat ML, Bozinovski S, Christie M (2021). Thermal ablation in non-small cell lung cancer: a review of treatment modalities and the evidence for combination with immune checkpoint inhibitors. Transl Lung Cancer Res.

[32] Streitparth T, Schumacher D, Damm R, Friebe B, Mohnike K, Kosiek O (2018). Percutaneous radiofrequency ablation in the treatment of pulmonary malignancies: efficacy, safety and predictive factors. Oncotarget.

[33] Palussière J, Cazayus M, Cousin S, Cabart M, Chomy F, Catena V (2021). Is there a role for percutaneous ablation for early stage Lung Cancer? What is the evidence?. Curr Oncol Rep.

[34] Huang BY, Li XM, Song XY, Zhou JJ, Shao Z, Yu ZQ et al. Long-term results of CT-guided percutaneous radiofrequency ablation of inoperable patients with stage Ia non-small cell lung cancer: A retrospective cohort study. Int J Surg. 2018; 53(143 – 50. 10.1016/j.ijsu.2018.03.034 10.1016/j.ijsu.2018.03.034 29555533

[35] Li M, Qin Y, Mei A, Wang C, Fan L (2020). Effectiveness of radiofrequency ablation therapy for patients with unresected stage IA non-small cell lung cancer. J Cancer Res Ther.

